# Comments on “On the significance of peak dose in normal tissue toxicity in spatially fractionated radiotherapy: The case of proton minibeam radiation therapy”

**DOI:** 10.1002/pro6.70026

**Published:** 2025-08-04

**Authors:** Zhaoning Wang, Linlin Wang

**Affiliations:** ^1^ Shandong Cancer Hospital and Institute Shandong First Medical University and Shandong Academy of Medical Sciences People's Republic of China

1

Dear Editor,

We read with interest the article, titled “On the significance of peak dose in normal tissue toxicity in spatially fractionated radiotherapy: The case of proton minibeam radiation therapy,” by Prezado et al.^1^ Compared with previous studies,^2^ this study addresses an often‐overlooked factor in spatially fractionated radiotherapy (SFRT): the impact of peak dose on normal tissue toxicity.

The novelty of this work is the focus on “peak dose,” which has largely been excluded compared to valley dose in previous studies.^3^, ^4^ By consistently maintaining average and valley doses, while varying only the peak dose, Prezado et al.^1^ experimentally demonstrated the impact of the peak dose on normal tissues, challenging established hypotheses in radiotherapy.

The study design is robust. Two distinct collimator configurations yielded peak doses of 55 Gy and 70 Gy, ensuring statistical verification of the experimental variables. Multiple behavioral assessments, such as open field, rotarod performance, object recognition, and Morris water maze, were used to evaluate motor, cognitive, and emotional functions, respectively, in rats. Histopathological analysis corroborated these behavioral observations. These findings suggested that even with unchanged average and valley doses, peak dose intensely affects tissue toxicity.

While this investigation offers valuable insights, several limitations warrant consideration. First, the sample size was relatively small (eight rats per group with some dropouts, which undermines the statistical power of the study hypothesis). Larger cohorts may facilitate better justification of these conclusions.^5^ Second, the study compared only two peak dose levels. Inclusion of intermediate dose groups in arithmetic progression might illustrate the dose‐toxicity relationship and help identify tolerance thresholds.^6^ More importantly, the study lacks assessment of tumor controls. While the authors suggest that lower peak doses might benefit normal tissue sparing, the absence of data on tumor response and the notion that an “intermediate high peak‐to‐valley dose ratio may be more conducive to balancing tumor control and tissue protection” renders the results speculative. Without documented evidence from tumor‐bearing models, conclusions on the clinical utility remain unaddressed. Therefore, subsequent research should focus on optimizing treatment to improve tumor control while minimizing toxicity in normal tissues. Finally, translating results from rodent brain tissue to humans is invalidated. Given that this study was conducted in vitro and interspecies variations in radiation responses may limit the clinical applicability of these findings, further investigation in real‐world clinical settings is warranted.

Despite these limitations, the study identifies critical gaps in the current literature. Future research should explore a broader dose range, assess tumor control, and utilize larger cohorts to validate these preliminary findings. Upon validation, clinical protocols for proton minibeam radiation therapy (pMBRT) might shift from the conventional “peak‐dose dominance” to toward “controlling the peak dose while maintaining the valley dose,” representing a paradigm shift in current practice. The study also highlights that the coupling relationship between dosimetry and geometric parameters in SFRT and its derivative techniques, such as MBRT and GRID therapy, is highly complex. A precise understanding of their interaction will be crucial for optimizing personalized treatment and ensuring patient safety.

In summary, while Prezado et al. contribute to the literature on dose optimization in SFRT, the study's limitations particularly the lack of tumor data and limited dose ranges restrict its clinical significance. Further studies are warranted before translating bench to bedside is possible.

## AUTHOR CONTRIBUTIONS

**Zhaoning Wang**: Writing—original draft. **Linlin Wang**: Writing—review & editing.

## CONFLICT OF INTEREST STATEMENT

There are no conflicts of interest to declare.

## ETHICS STATEMENT

Not applicable.

## Data Availability

No datasets were generated or analysed during the study.
